# Preparation of Ni-Mn ferrites magnetic nanoparticles through the ethanol solution combustion-calcination process for the adsorption of methyl blue

**DOI:** 10.1371/journal.pone.0321741

**Published:** 2025-05-09

**Authors:** Zhongjun Pan, Zhou Wang, Zhixiang Lv

**Affiliations:** 1 The People’s Hospital of Danyang, Affiliated Danyang Hospital of Nantong University, Zhenjiang, P.R. China; 2 College of Vanadium and Titanium, Panzhihua University, Panzhihua, P.R. China; Brandeis University, UNITED STATES OF AMERICA

## Abstract

Ni-Mn ferrites magnetic nanoparticles (MNPs) were successfully prepared through the ethanol solution combustion-calcination process, and characterized by SEM, TEM, XRD, VSM, BET, and FTIR techniques. For smaller particle size and suitable magnetic property, the optimum element ratio of the material was Ni_0.9_Mn_0.1_Fe_2_O_4_, and the optimal preparation conditions were appropriate ethanol dosage to attain Fe^3+^ concentrations of approximately 0.85 M, calcination temperature of 400 °C, and calcination time of 2 h, their specific surface area was 136.5 m^2^/g, and their average particle size and saturation magnetization were 35 nm and 21.66 emu/g, respectively. The adsorption process of methyl blue (MB) onto Ni_0.9_Mn_0.1_Fe_2_O_4_ MNPs conformed to the pseudo-second-order adsorption kinetic model in the initial concentrations of 100–250 mg/L. In comparison with Langmuir and Freundlich adsorption isotherm models, the Temkin model (R^2^ = 0.9865) was observed to better demonstrate the state of MB onto Ni_0.9_Mn_0.1_Fe_2_O_4_ MNPs, revealing that the adsorption mechanism of MB onto Ni_0.9_Mn_0.1_Fe_2_O_4_ MNPs was the multi-molecular chemical process. The adsorption capacity of Ni_0.9_Mn_0.1_Fe_2_O_4_ MNPs for MB still maintained about 90% of the initial adsorbance after 6 times cyclic utilization of the nanoparticles by recalcination method, suggesting that Ni_0.9_Mn_0.1_Fe_2_O_4_ MNPs had excellent regeneration performance. In general, these results coupled with its environmental friendliness attributed the potential candidates for effluent remediation.

## 1. Introduction

The issue of pollution is increasingly becoming more severe, and has aroused human’s great attention, including water and atmospheric pollution, soil degradation, noise disturbance, and other forms of pollution [1–4]. Among these concerns, water pollution has gained increasing prominence due to the extreme scarcity of freshwater resources [5,6]. Especially, dye-containing effluent attaches great importance as it has posed a grave threat on water environment and human health, even human life, owing to its high toxicity, chemical stability, and slow degradation rate [7,8]. However, according to statistics, about 80% of the dye effluent is discharged into the environment without treatment [9].

The treatment of these effluents has been listed as the focus of environmental protection. An effective measure is badly necessitated. A variety of techniques have been developed to eliminate dye-based pollutants from water solutions, including physical method [10], chemical process [11–13], biological process [14,15], and so on. Among them, as the physical method, adsorption has garnered significant attention due to its cost-effectiveness and superior efficiency [16,17]. Additionally, the chemical decomposition or transformation of pollutants is not presented in the adsorption process, thus avoiding the generation of new contaminants. What’s more, the adsorption process occurs the surface of adsorbents, including outside surface and internal surface, thus the adsorbents play a significant pivotal role [18].

At present, the commonly used adsorbents include activated carbon, polymer resin and mineral adsorbents, etc., but they all have certain limitations, such as difficulty in regeneration, operational complexity and, most importantly, the limitation in adsorption capacity [19,20]. Compared with conventional adsorbents, nanoparticles have attracted much more attention owing to their large specific surface area and large adsorption capacity [21]. However, practical applications can be inconvenienced by the difficulty of collecting nanoparticles from their dispersing media. In this context, magnetic nanoparticles, which can be easily recycled with an external magnetic field [22], thus facilitating the application and cost management of magnetic nanoparticles while minimizing the number of material syntheses [23–25], are one of the desirable candidate adsorbents for effluent treatment. Particularly, spinel ferrites (MFe_2_O_4_) nanoparticles, providing excellent magnetic susceptibility, functionalization potential, low cost as well as unique advantages in regenerating and recycling, are among the most promising of these compounds [26].

There are many preparation methods for magnetic ferrite-based nanoparticles, such as precipitation, sol-gel, hydrothermal, high-energy ball milling [27,28], etc. As a new preparation method, the ethanol solution combustion-calcination process has the advantages of simple work-up procedure, shorter reaction time and preparation cycle, low cost, environmental friendliness, low requirements on the equipment, easy realization of industrialized production, and so on.

For the purpose of developing a nanomaterial with high adsorption capacity and satisfactory recycling performance, Ni_0.9_Mn_0.1_Fe_2_O_4_ MNPs prepared through the ethanol solution combustion-calcination process were selected and employed to remove dyes in this work, and with methyl blue (MB) as adsorbate model, the adsorption performance of the azo dyes on Ni_x_Mn_(1-x)_Fe_2_O_4_ MNPs from water solution was explored [29,30].

## 2. Experiments

### 2.1. *Preparation and characterization of Ni-Mn ferrites MNPs*

Ni_x_Mn_(1-x)_Fe_2_O_4_ MNPs were prepared through the ethanol solution combustion-calcination process using absolute ethanol as solvent and fuel. Fe(NO_3_)_3_·9H_2_O, Ni(NO_3_)_2_·6H_2_O, and Mn(NO_3_)_2_·4H_2_O were precisely weighed according to the stoichiometric ratio, and dissolved in a beaker with anhydrous ethanol as a solvent to achieve Fe^3+^ concentrations of about 0.85 M, 0.57 M, 0.43 M, 0.34 M, and 0.17 M, respectively. When all the nitrates were completely dissolved to form homogeneous solutions, the solutions were transferred into crucibles and ignited. After the flames were extinguished, the crucibles together with intermediates were calcined at various temperatures (400 °C, 500 °C, 600 °C, and 700 °C) for 2 h. Finally, the calcined products were placed in a mortar and ground to form powders.

The phase identification of Ni-Mn ferrites MNPs was measured by XRD and FTIR, the morphology was investigated with SEM and TEM, the chemical composition was detected by EDS, the magnetic measurement was examined by VSM, and the specific surface area was measured by BET method.

### 2.2. *Adsorption of MB onto Ni*_*0.9*_*Mn*_*0.1*_*Fe*_*2*_*O*_*4*_ *MNPs*

Ni_0.9_Mn_0.1_Fe_2_O_4_ MNPs were selected to remove MB. At ambient temperature, adsorption kinetics experiments were performed by keeping 5 mg of Ni_0.9_Mn_0.1_Fe_2_O_4_ MNPs in a series of centrifuge tubes containing 2 mL MB solution with initial concentrations (100, 150, 200, and 250 mg/L). The adsorbents were subsequently separated at regular intervals (10–180 min), and the adsorption capacities of Ni_0.9_Mn_0.1_Fe_2_O_4_ MNPs were determined based on the change of MB concentration.

The adsorption isotherm of MB onto the nanoparticles was investigated by the similar method, 10 different initial concentrations of MB solution ranging from 400 mg/mL to 4000 mg/mL were adsorbed for 24 h. Subsequently, the supernatants of different initial concentrations were measured.

Additionally, to explore the influence of pH on adsorbance of MB onto Ni_0.9_Mn_0.1_Fe_2_O_4_ MNPs, the pH values of 1, 3, 5, 7, 9, 11, and 13 were adjusted by 1 M dilute HCl or dilute NaOH solutions of, and the adsorption capacities under various pH values were obtained. As to the cycle capacity of Ni_0.9_Mn_0.1_Fe_2_O_4_ MNPs [31], 75 mg Ni_0.9_Mn_0.1_Fe_2_O_4_ MNPs were dispersed into 30 mL MB of 500 mg/L in Erlenmeyer flask for 3 h. Upon completion of the adsorption, Ni_0.9_Mn_0.1_Fe_2_O_4_ MNPs were centrifuged and recalcined at 400 °C for 2 h to regenerate the nanoparticles. The experiment described above was replicated after grinding and the adsorption process of MB onto Ni_0.9_Mn_0.1_Fe_2_O_4_ MNPs was verified by infrared spectroscopy.

The absorbances of MB solutions were detected using a UV-Vis spectrophotometer at 600 nm, and the corresponding concentrations were obtained according to the relationship of the absorbance and MB concentration; while, the adsorbance of MB loaded onto Ni_0.9_Mn_0.1_Fe_2_O_4_ MNPs was calculated Using Eq. (1) [32].


$$q_{e}=\frac{V(C_{0}-C_{e})}{m}$$ (1)

Wherein, *q*_*e*_ represented the equilibrium adsorbance of MB onto Ni_0.9_Mn_0.1_Fe_2_O_4_ MNPs; *m* was the mass of Ni_0.9_Mn_0.1_Fe_2_O_4_ MNPs; *V* indicated the volume of MB solution; *C*_*0*_ and *C*_*e*_ were the initial and equilibrium concentrations [18].

## 3. Results and discussion

### 3.1. *Characteristics of Ni*_*0.9*_*Mn*_*0.1*_*Fe*_*2*_*O*_*4*_ *MNPs*

**Fig 1:**
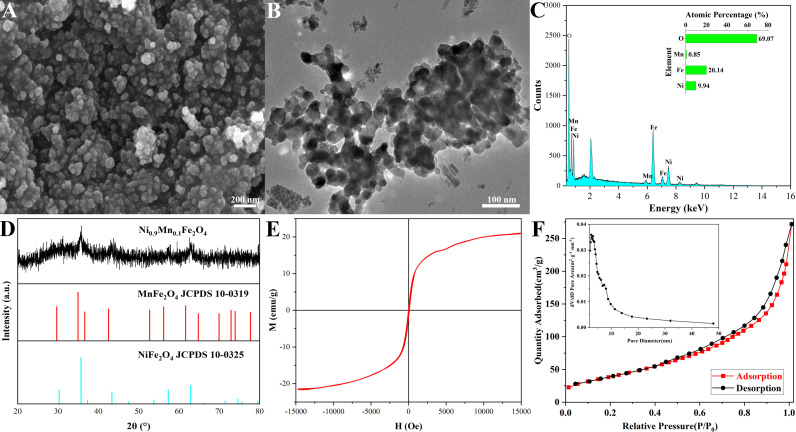
SEM morphology (A), TEM image (B), EDS spectrum (C), XRD pattern (D), the hysteresis loops (E), and N_2_ sorption isotherm (F) of Ni_0.9_Mn_0.1_Fe_2_O_4_ MNPs calcined at 400 °C with 20 mL ethanol and Fe^3+^ concentrations of 0.85 M.

Fig 1 showed the characteristics of Ni_0.9_Mn_0.1_Fe_2_O_4_ MNPs calcined at 400 °C with 20 mL ethanol to achieve a Fe^3+^ concentration of 0.85 M. The SEM morphology (Fig 1A/S1 Fig) displayed that their average particle size was about 35 nm, and the distribution of the particle sizes was uniform. The TEM image (Fig 1B/S2 Fig) suggested that the average particle size of Ni_0.9_Mn_0.1_Fe_2_O_4_ MNPs was also approximately 35 nm, which was consistent with the result from SEM morphology. The EDS spectrum was displayed in Fig 1C, the atomic percentages of Ni, Mn, Fe and O in Ni_0.9_Mn_0.1_Fe_2_O_4_ MNPs were basically consistent with the designed composition, which revealed the successful preparation of magnetic Ni_0.9_Mn_0.1_Fe_2_O_4_ nanoparticles. The crystal structures of the Ni_0.9_Mn_0.1_Fe_2_O_4_ nanoparticles were investigated by XRD. Fig 1D illustrated that the based diffraction peaks of Ni_0.9_Mn_0.1_Fe_2_O_4_ MNPs could be indexed to the standard NiFe_2_O_4_ PDF card (JCPDS No. 10–0325) and MnFe_2_O_4_ PDF card (JCPDS No. 10–0319). Fig 1E displayed the hysteresis loops of Ni_0.9_Mn_0.1_Fe_2_O_4_ MNPs with typical soft magnetic characteristic, their saturation magnetization (Ms) was about 21.66 emu/g, which demonstrated the superparamagnetism of the material [25,33]. All these results indicated that Ni_0.9_Mn_0.1_Fe_2_O_4_ MNPs were successfully prepared. Fig 1F showed the N_2_ sorption isotherm of Ni_0.9_Mn_0.1_Fe_2_O_4_ MNPs, the adsorption-desorption isotherm curve belonged to the type of Ⅳ, and an obvious hysteresis ring appeared, the specific surface area of Ni_0.9_Mn_0.1_Fe_2_O_4_ MNPs was 136.5 m^2^/g, and their average pore size distributed from 2 nm to 10 nm, the numerous inner bores contributed to large specific surface area, and the large specific surface area was the important factor for the large adsorption capacity of Ni_0.9_Mn_0.1_Fe_2_O_4_ MNPs.

### 3.2. *Optimization of preparation process for Ni*_*x*_*Mn*_*(1-x)*_*Fe*_*2*_*O*_*4*_ *MNPs*

#### 3.2.1. *Influence of element proportion.*

**Fig 2:**
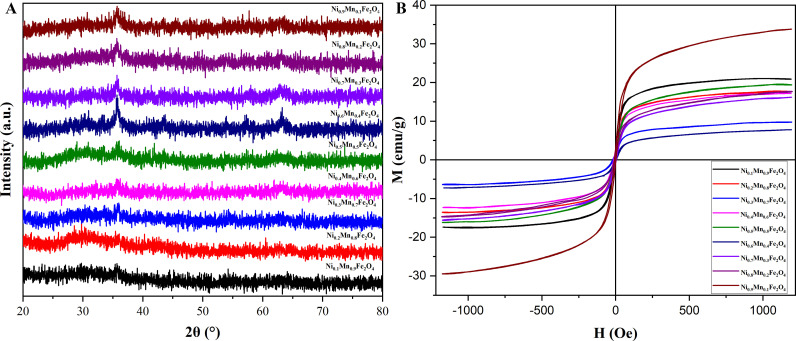
XRD pattern (A) and hysteresis loops (B) of Ni_x_Mn_(1-x)_Fe_2_O_4_ (x = 0.1–0.9) MNPs with different element ratios.

It could be seen from Fig 2A that when x = 0.1–0.5, there were no obvious characteristic peaks in the spectra, which might ascribe to the fact that the nanoparticles failed to form stable crystallization at this time, and was disregarded. When x = 0.6–0.9, there were obvious characteristic peaks. When x = 0.9, the characteristic peak was the widest, suggesting that the crystallinity was the lowest, and their average particle size of Ni_x_Mn_(1-x)_Fe_2_O_4_ MNPs was the smallest. Fig 2B showed the hysteresis loops obtained by measuring the performance of Ni_x_Mn_(1-x)_Fe_2_O_4_ (x = 0.1–0.9) MNPs by vibrating sample magnetometer. The saturation magnetization of magnetic Ni_0.9_Mn_0.1_Fe_2_O_4_ nanoparticles was the highest of 33.80 emu/g, which provided a guarantee for the separation and recovery of Ni_0.9_Mn_0.1_Fe_2_O_4_ nanoparticles.

#### 3.2.2. *Effects of calcination temperature and ethanol dosage.*

**Fig 3:**
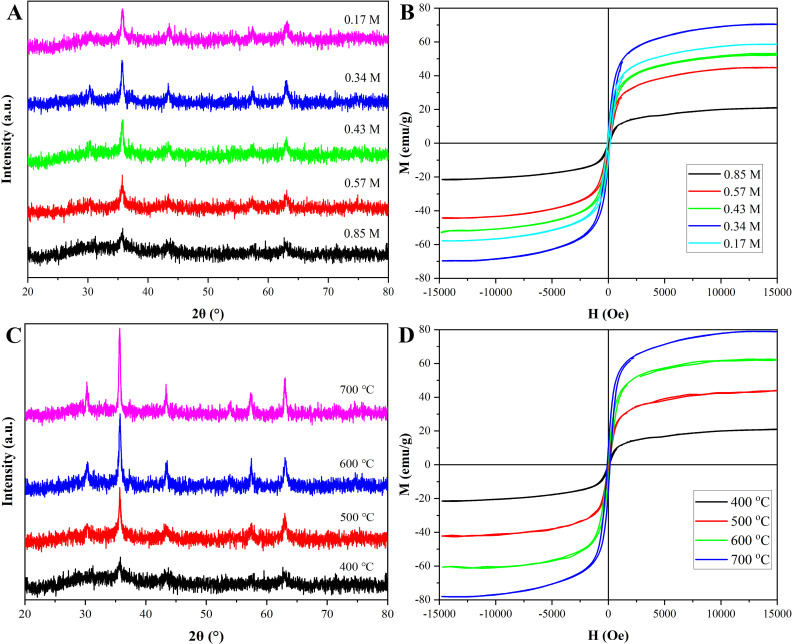
XRD patterns (A, B) and hysteresis loops (C, D) of Ni_0.9_Mn_0.1_Fe_2_O_4_ MNPs prepared with different Fe^3+^ concentrations and at various temperatures.

Fig 3A revealed the XRD patterns of Ni_0.9_Mn_0.1_Fe_2_O_4_ MNPs prepared at 400 °C for 2 h with various ethanol volumes for Fe^3+^ concentrations of about 0.85 M, 0.57 M, 0.43 M, 0.34 M, and 0.17 M, respectively. The change of the ethanol dosage would affect the combustion time and the dispersion degree of the preparation process, thus affect the grain size. As was well-known, the long combustion time could increase the degree of crystallinity, and the large dispersion degree of mental ions could reduce the degree of crystallinity. At low doses of ethanol solvent, i.e., large Fe^3+^ concentrations, the combustion time of the ingredient solution was short, and the dispersion degree of mental ions was low, but the combustion time played a mainly larger effect; therefore, with Fe^3+^ concentrations changing from 0.85 M to 0.34 M, the degree of crystallinity increased, and the diffraction peak became narrower and higher. However, with Fe^3+^ concentrations reaching 0.17 M, the dispersion degree of the mental ions began to play the larger effect, resulting in the decrease of the crystallinity degree, therefore, the diffraction peak became shorter and wider, and the average particle size increased. Fig 3B showed the hysteresis loops of Ni_0.9_Mn_0.1_Fe_2_O_4_ MNPs with different concentrations of ingredient solutions. Obviously, the nanoparticles retained excellent magnetic properties even at low doses (i.e., Fe^3+^ concentration of 0.85 M). With the decrease of Fe^3+^ concentration, the degree of crystallinity increased, Ms of Ni_0.9_Mn_0.1_Fe_2_O_4_ MNPs increased. When Fe^3+^ concentration reached 0.17 M, the degree of crystallinity decreased, Ms of Ni_0.9_Mn_0.1_Fe_2_O_4_ MNPs also decreased [34]. Based on the above analysis, to obtain the nanoparticles with larger adsorption performance, the Fe^3+^ concentration of the raw material solution for the preparation of Ni_0.9_Mn_0.1_Fe_2_O_4_ MNPs was selected as 0.85 M.

Fig 3C displayed the XRD patterns of Ni_0.9_Mn_0.1_Fe_2_O_4_ MNPs calcined at 400 °C, 500 °C, 600 °C, and 700 °C for 2 h with Fe^3+^ concentration of 0.85 M. The diagram indicated that the characteristic peaks were more prominent with the rise of calcination temperature. When the calcination temperature was 400 °C, the characteristic peaks were the widest and the lowest. Too high temperature would cause the material to agglomerate and increase the particle size, resulting in the decreases of the specific surface area owing to the decrease of inner bore, and finally a descent of the adsorbance. Moreover, with the increase of calcination temperature, the pores inside the nanoparticles would collapse, which would also lead to poor adsorption performance. Fig 3D displayed the hysteresis loops of Ni_0.9_Mn_0.1_Fe_2_O_4_ MNPs calcined at different temperatures, and the saturation magnetization strength of the nanoparticles similarly increased with the rise of calcination temperature. Although higher temperatures leaded to better magnetic properties, they also leaded to too large nanoparticle size, which made adsorption capacity decrease. Considering comprehensively, the sample calcined at 400 °C should be selected for the next experiments.

### 3.3. *Adsorption of MB onto Ni*_*0.9*_*Mn*_*0.1*_*Fe*_*2*_*O*_*4*_ *MNPs*

#### 3.3.1. *Adsorption kinetics.*

To explore the adsorption mechanism, different models were employed to fit the adsorption data, and the adsorbance of MB onto Ni_0.9_Mn_0.1_Fe_2_O_4_ MNPs with time was investigated by fitting the curve and kinetics parameters. For this assessment, the pseudo-first-order, pseudo-second-order and intraparticle diffusion models as expressed in Eq. (2) [35], Eq. (3) [18] and Eq. (4) [36] were applied.


$$q_{t}\text{ = }q_{e}(1-e^{k_{1}t})$$ (2)

$$q_{t}=\frac{{q_{e}}^{2}k_{2}t}{1+q_{e}k_{2}t}$$ (3)

$$q_{t}=x_{i}+k_{i}t^{1/2}$$ (4)

Wherein, *q*_*e*_ and *q*_*t*_ were the adsorbances of MB onto Ni_0.9_Mn_0.1_Fe_2_O_4_ MNPs at equilibrium time and a given time; *k*_*1*_, *k*_*2*_ and *k*_*i*_ were the rate constants for three models; *x*_*i*_ was associated to the thickness of boundary layer.

**Fig 4:**
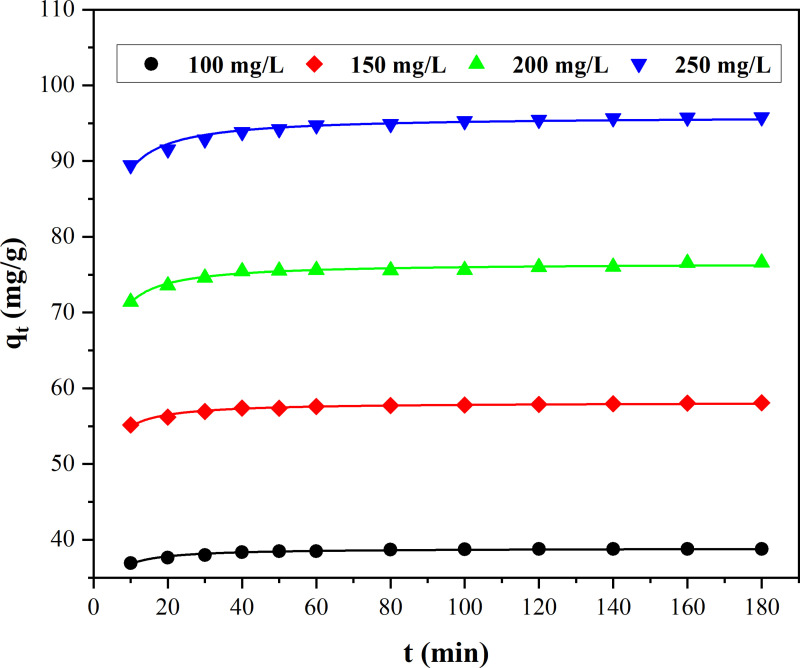
Adsorption process of MB onto Ni_0.9_Mn_0.1_Fe_2_O_4_ MNPs for various initial MB concentrations at room temperature.

**Fig 5:**
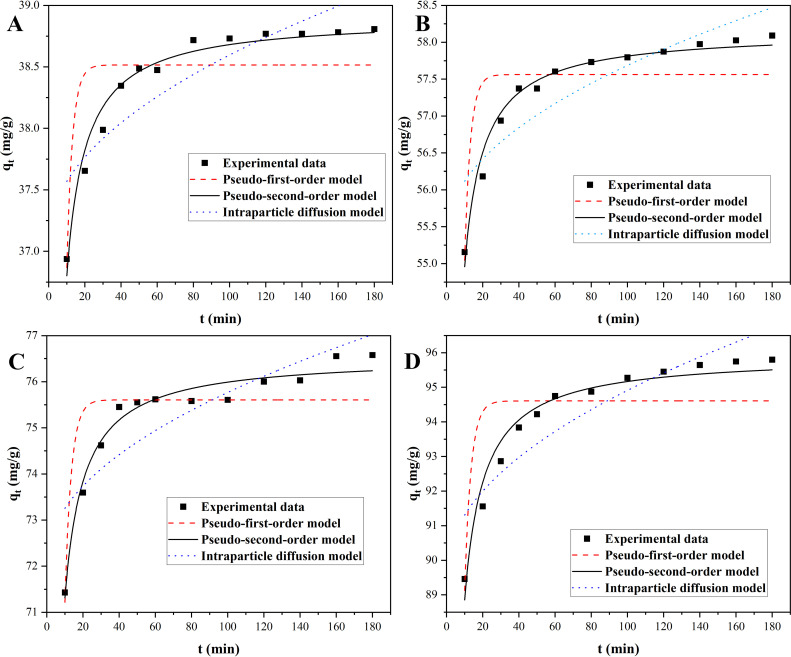
The fitting curves for three kinetics models with initial MB concentrations of 100 mg/L (A), 150 mg/L (B), 200 mg/L (C), and 250 mg/L (D).

**Fig 6:**
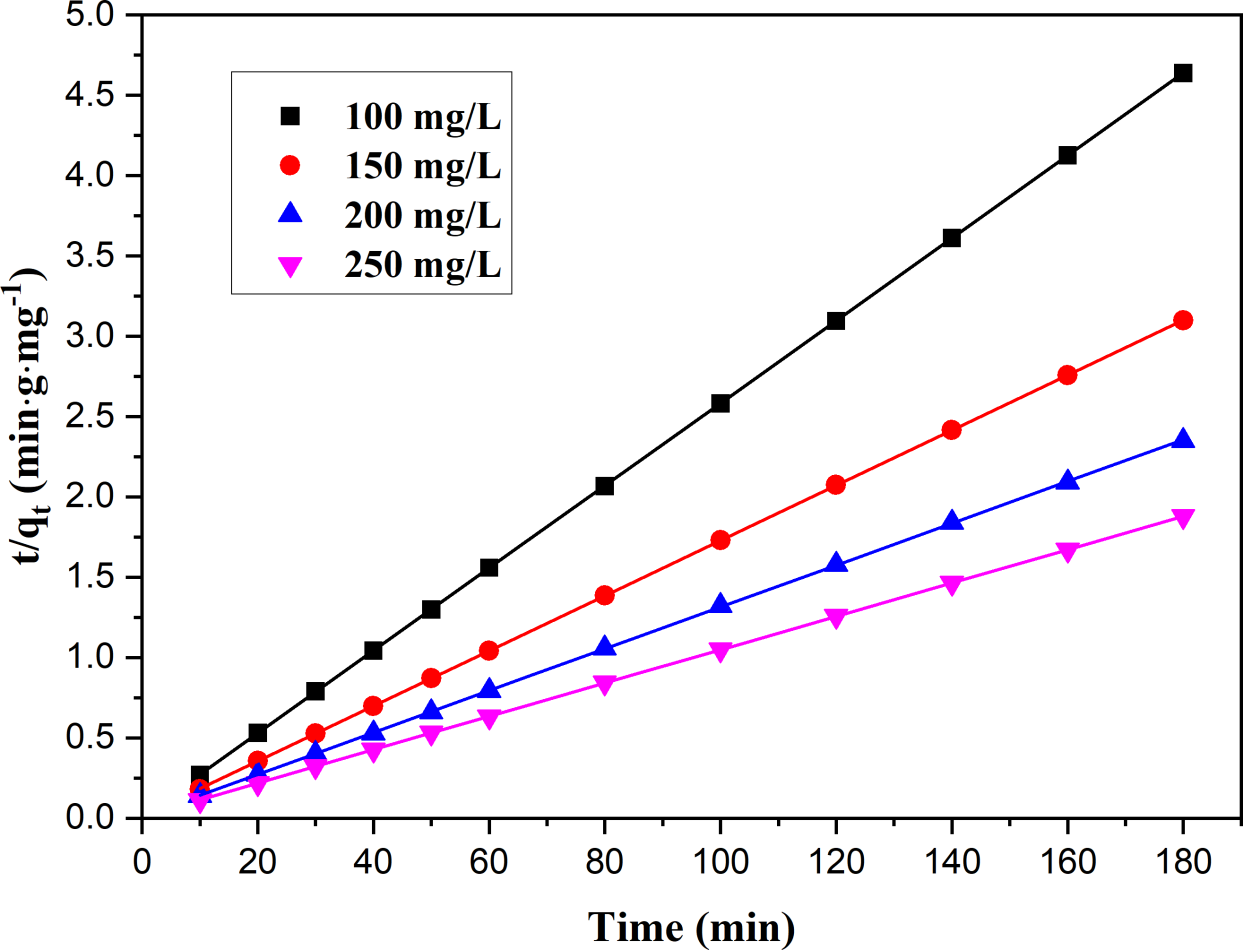
Plots of *t*/*q*_t_ versus *t* for adsorption of MB onto magnetic Ni_0.9_Mn_0.1_Fe_2_O_4_ MNPs with the various initial concentrations at room temperature.

The experimental curves for the adsorption of MB onto Ni_0.9_Mn_0.1_Fe_2_O_4_ MNPs with various initial MB concentrations at room temperature with time were revealed in Fig 4. The adsorbances of MB onto Ni_0.9_Mn_0.1_Fe_2_O_4_ MNPs rose with the increase of the initial MB concentration. Under the same concentration, the adsorption rate was fast followed by slow until the equilibrium was reached. The fitted curves with three kinetic models for MB onto Ni_0.9_Mn_0.1_Fe_2_O_4_ MNPs were presented in Fig 5, and all the fitted kinetic parameters were listed in Table 1. The pseudo-second-order kinetics model showed the best fitting based on the variances (R^2^) for three models, and their variances were larger than 0.96, and the line relationships of the pseudo second-order adsorption kinetic model for the adsorption of MB onto Ni_0.9_Mn_0.1_Fe_2_O_4_ MNPs with various MB initial concentrations were displayed in Fig 6, which revealed better line correlations, all the results suggested that the adsorption of MB onto Ni_0.9_Mn_0.1_Fe_2_O_4_ MNPs might be a chemical adsorption involving electron sharing or electron transfer.

**Table 1:** The simulative adsorption kinetics parameters for MB onto Ni_0.9_Mn_0.1_Fe_2_O_4_ MNPs at room temperature.

Adsorption kinetics model	parameter	initial concentrations of MB (mg/L)			
		100	150	200	250
pseudo-first-order kinetics	R^2^	0.6432	0.6523	0.7252	0.6097
	K_1_	0.3151	0.3129	0.2846	0.2848
	Adj. R^2^	0.6076	0.6176	0.6977	0.5707
pseudo-second-order kinetics	R^2^	0.9776	0.9760	0.9710	0.9625
	K_2_	0.0450	0.0296	0.0178	0.0131
	Adj. R^2^	0.9695	0.9737	0.9682	0.9587
internal diffusion	R^2^	0.7521	0.7638	0.7212	0.8120
	K_i_	0.1502	0.2295	0.3674	0.5272
	Adj. R^2^	0.7274	0.7402	0.6933	0.7932

#### 3.3.2. *Adsorption isotherms.*

The adsorption isotherm could provide the interaction relationship of MB molecules and the state of MB on the surfaces of Ni_0.9_Mn_0.1_Fe_2_O_4_ MNPs. Therefore, Langmuir, Freundlich, and Temkin adsorption isotherm models were employed to simulate the adsorption equilibrium curves.

Langmuir model could be described by the Eq. (5) [37].


$$q_{e}=\frac{q_{\max}K_{L}C_{e}}{1+K_{L}C_{e}}$$ (5)

wherein *q*_*e*_ and *q*_*max*_ were the equilibrium and the maximum adsorbances of MB onto Ni_0.9_Mn_0.1_Fe_2_O_4_ MNPs, C_e_ was the equilibrium concentration of MB, and K_L_ was the rate of adsorption.

Freundlich model assumed that the adsorption of adsorbate onto adsorbent was bistratal, and its equation was described by Eq. (6) [30].


$$q_{e}=K_{F}C_{e}^{\frac{1}{n}}$$ (6)

Whereas K_F_ was a constant for Freundlich model. 1/n was a dimensionless factor that reflected the adsorption intensity or surface heterogeneity.

Temkin model assumed that the adsorption of adsorbate onto adsorbent was tanglesome, and its expression was shown as Eq. (7) [38].


$$q_{e}=B\;\text{In}(A_{T}C_{e})$$ (7)

Wherein, B and A_T_ were the constant for Temkin and the equilibrium binding constant, respectively.

**Fig 7:**
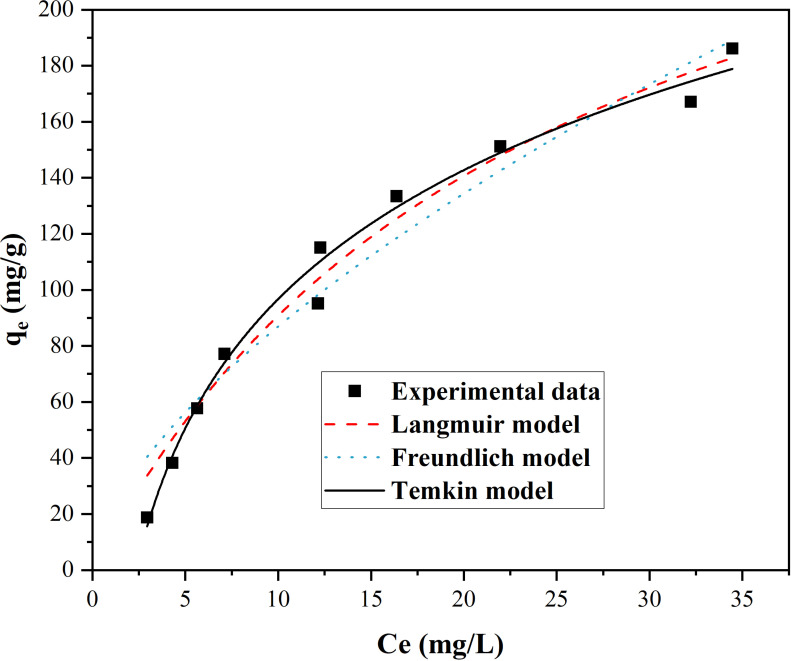
Adsorption isotherm of MB onto Ni_0.9_Mn_0.1_Fe_2_O_4_ MNPs at ambient temperature.

Nonlinear regression methods were utilized to fit the equilibrium data and evaluate the parameters associated with these models, and the fitting curves were displayed in Fig 7, and the simulative parameters were demonstrated in Table 2. Comparing their R^2^, Temkin model acquired the best fitting, and its R^2^ value reached 0.9865, which indicated that Temkin model was most suitable for explain the adsorption state of MB onto Ni_0.9_Mn_0.1_Fe_2_O_4_ MNPs. According to the theory of Temkin isothermal model, the adsorption of MB onto Ni_0.9_Mn_0.1_Fe_2_O_4_ MNPs should belong to multi-molecular layer chemical adsorption mechanism [39].

**Table 2:** Simulative parameters of adsorption isotherms for MB onto Ni_0.9_Mn_0.1_Fe_2_O_4_ MNPs at room temperature.

adsorption isotherm model	R^2^	fitting result	parameter	parameter value
Langmuir	0.9746	0.9714	q_max_	312.0523
			K_L_	0.0400
Freundlich	0.9484	0.9419	K_F_	20.4684
			1/*n*	0.6281
Temkin	0.9865	0.9848	B_T_	66.4165
			A_T_	0.4287

#### 3.3.3. *Effect of pH on adsorbance and regeneration study.*

**Fig 8:**
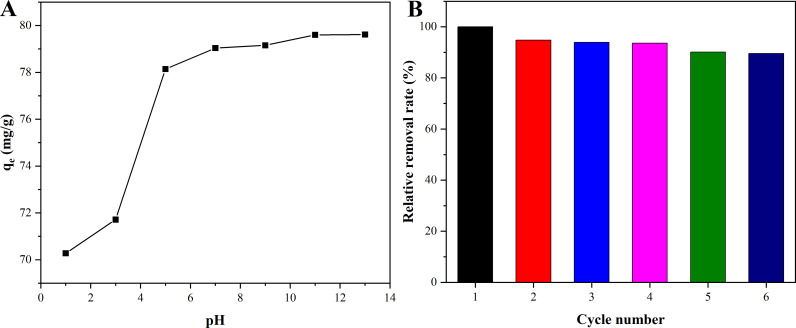
Influence of pH on the adsorbance of MB onto Ni_0.9_Mn_0.1_Fe_2_O_4_ MNP at room temperature (A) and the regeneration of Ni_0.9_Mn_0.1_Fe_2_O_4_ MNPs (B).

The pH of MB solution had significant impact on the adsorption, because pH not only affected the surface charge of Ni_0.9_Mn_0.1_Fe_2_O_4_ MNPs, but also affected the property of the dye itself [40]. To reveal the influence of pH on the adsorbance of MB onto Ni_0.9_Mn_0.1_Fe_2_O_4_ MNPs, the experiments were carried out with various pH of MB solution and the initial MB concentration of 200 mg/L, and the effect of pH on the adsorption was displayed in Fig 8A. When the pH exceeded 5, the adsorption capacity of MB onto Ni_0.9_Mn_0.1_Fe_2_O_4_ MNPs remained a large value. However, once the pH was less than 5, the adsorption capacity decreased sharply. The reason for this phenomenon was that the surface of Ni_0.9_Mn_0.1_Fe_2_O_4_ MNPs was positively charged owing to MB isoelectric point of about 5. When the pH was less than 5, the surface of MB was positively charged, and there was an electrostatic repulsion between MB and Ni_0.9_Mn_0.1_Fe_2_O_4_ MNPs, which gradually decreased with the increase of pH value. As the pH was greater than 5, MB was negatively charged, the dissimilar electrostatics resulted in combination of MB and Ni_0.9_Mn_0.1_Fe_2_O_4_ MNPs, and the adsorption capacity reached the maximum, and did not change, suggesting that the saturated adsorption state had been reached at this time. In short, the nanoparticles could maintain a large adsorption capacity for MB in a large pH range from 5 to 13.

The regeneration of Ni_0.9_Mn_0.1_Fe_2_O_4_ MNPs was shown in Fig 8B. With the increase in reuse frequency of Ni_0.9_Mn_0.1_Fe_2_O_4_ MNPs, the adsorbance of MB onto Ni_0.9_Mn_0.1_Fe_2_O_4_ MNPs gradually reduced. This might be attributed to the fact that the repeated calcination process increased the sintering degree of Ni_0.9_Mn_0.1_Fe_2_O_4_ MNPs and the proportion of pore collapse, resulting in a decrease of their specific surface area. Importantly, after 6 rounds of regeneration, the adsorbance of MB onto Ni_0.9_Mn_0.1_Fe_2_O_4_ MNPs still maintained about 90% of the first adsorption capacity, indicating that Ni_0.9_Mn_0.1_Fe_2_O_4_ MNPs had excellent recycling performance.

The congeneric adsorbents related for MB adsorptions were summarized and were listed in Table 3. Compared with the congeneric adsorbents, including Co_0.5_Zn_0.5_Fe_2_O_4_ MNPs, MnFe_2_O_4_ MNRs, Co_0.8_Cu_0.2_Fe_2_O_4_ MNPs, Co_0.4_Cu_0.2_Zn_0.4_Fe_2_O_4_ MNPs, Mg_0.5_Cu_0.5_Fe_2_O_4_ MNPs, the adsorption capacity of MB onto Ni_0.9_Mn_0.1_Fe_2_O_4_ MNPs was almost 1.65 3.05 times of ones for the congeneric adsorbents, suggesting the promising application of Ni_0.9_Mn_0.1_Fe_2_O_4_ MNPs for the removal of MB.

**Table 3:** Comparison of the congeneric adsorbents for MB adsorptions.

Adsorbent	Adsorption capacity (mg/g)	Reference
Co_0.5_Zn_0.5_Fe_2_O_4_ MNPs	189.1	[41]
MnFe_2_O_4_ MNRs	102.3	[42]
Co_0.8_Cu_0.2_Fe_2_O_4_ MNPs	174.2	[43]
Co_0.4_Cu_0.2_Zn_0.4_Fe_2_O_4_ MNPs	140.4	[44]
Mg_0.5_Cu_0.5_Fe_2_O_4_ MNPs	123.8	[45]
Ni_0.9_Mn_0.1_Fe_2_O_4_ MNPs	312.1	This work

#### 3.3.4. *FTIR spectrum.*

**Fig 9:**
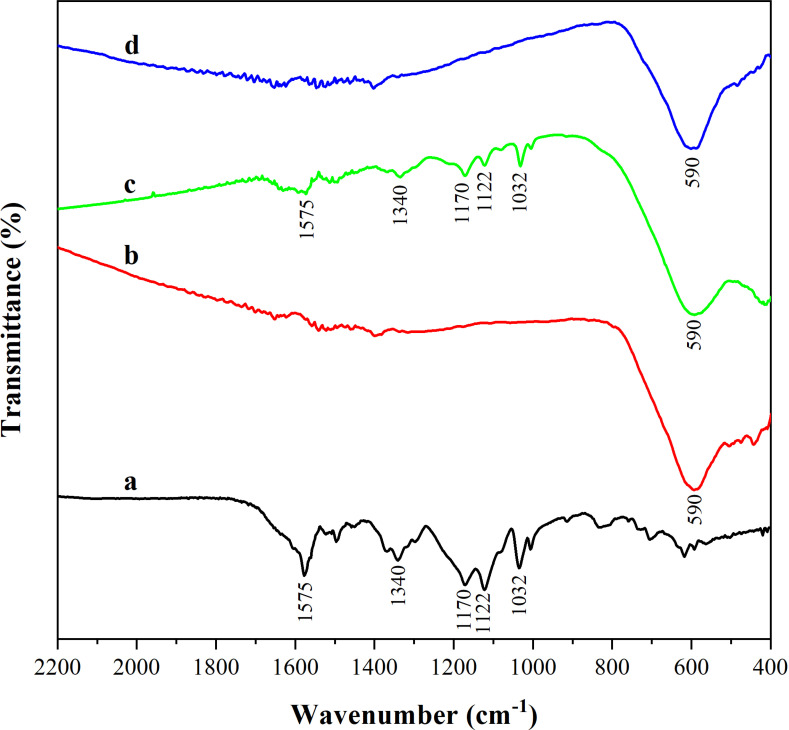
FTIR spectrum of MB (A), Ni_0.9_Mn_0.1_Fe_2_O_4_ MNPs (B), Ni_0.9_Mn_0.1_Fe_2_O_4_-MB (C) and Ni_0.9_Mn_0.1_Fe_2_O_4_ MNPs recalcined at 400 °C for 2 h after adsorption (D).

FTIR spectroscopy was used to indicate the reproductive property of Ni_0.9_Mn_0.1_Fe_2_O_4_ MNPs. The infrared spectra of MB (Fig 9A), Ni_0.9_Mn_0.1_Fe_2_O_4_ MNPs (Fig 9B), Ni_0.9_Mn_0.1_Fe_2_O_4_ MNPs after adsorption of MB (Fig 9C), and Ni_0.9_Mn_0.1_Fe_2_O_4_ MNPs recalcined at 400 °C for 2 h after adsorption (Fig 9D) were analyzed. As shown in the figure, there are five obvious characteristic peaks of 1032 cm^-1^,1122 cm^-1^,1170 cm^-1^,1340 cm^-1^, and 1575 cm^-1^ on the infrared spectrum of MB. Compared with Ni_0.9_Mn_0.1_Fe_2_O_4_ MNPs unadsorbed MB, the infrared spectrum of the adsorbed Ni_0.9_Mn_0.1_Fe_2_O_4_ MNPs showed five characteristic peaks corresponding to MB in addition to the Fe-O characteristic peak at 590 cm^-1^, which verified that MB was successfully adsorbed onto Ni_0.9_Mn_0.1_Fe_2_O_4_ MNPs. When Ni_0.9_Mn_0.1_Fe_2_O_4_ MNPs adsorbed MB were calcined at 400 °C for 2 h, the characteristic peak of MB in the infrared spectrum basically disappeared and meanwhile the characteristic peak of Ni_0.9_Mn_0.1_Fe_2_O_4_ MNPs reappeared, demonstrating that the regeneration of Ni_0.9_Mn_0.1_Fe_2_O_4_ MNPs could be realized after calcination.

## 4. Conclusions

(1) Ni_x_Mn_(1-x)_Fe_2_O_4_ MNPs were prepared through the ethanol solution combustion-calcination process, and the as-prepared Ni_0.9_Mn_0.1_Fe_2_O_4_ MNPs under optimum preparation conditions (Fe^3+^ concentrations of approximately 0.85 M, the calcination temperature of 400 °C, calcination time of 2 h) with average diameter of about 35 nm, Ms of 21.66 emu/g, and the specific surface area of 136.5 m^2^/g were selected for the removal of MB.
(2) Pseudo-second-order kinetic and Temkin models were the best fittings for the adsorption data of MB onto Ni_0.9_Mn_0.1_Fe_2_O_4_ MNPs at ambient temperature, indicating that the multi-molecular layer chemisorption process involving electron sharing or electron transfer was more likely to be the principal underlying mechanism for the removal of MB.
(3) The influence of pH on the adsorbance showed that the adsorption of MB on Ni_0.9_Mn_0.1_Fe_2_O_4_ MNPs could maintain a large adsorption capacity for MB in a range of pH > 5. The FTIR spectroscopy and regeneration performance proved that Ni_0.9_Mn_0.1_Fe_2_O_4_ MNPs could be regenerated by calcination, and still maintain a high adsorption capacity even regenerated for 6 times, exhibiting the excellent reusability and stability of Ni_0.9_Mn_0.1_Fe_2_O_4_ MNPs.

## Supporting information

S1 Fig SEM morphology of Ni_0.9_Mn_0.1_Fe_2_O_4_ MNPs calcined at 400 °C with 20 mL ethanol and Fe^3+^ concentrations of 0.85 M. (TIF)

S2 Fig TEM image of Ni_0.9_Mn_0.1_Fe_2_O_4_ MNPs calcined at 400 °C with 20 mL ethanol and Fe^3+^ concentrations of 0.85 M. (BMP)
