# Supplementary figures and images for: Preparation of Ni-Mn ferrites magnetic nanoparticles through the ethanol solution combustion-calcination process for the adsorption of methyl blue
Source: PLoS One. 2025 May 9;20(5):e0321741. doi: 10.1371/journal.pone.0321741 (PMC12063861; doi:10.1371/journal.pone.0321741)

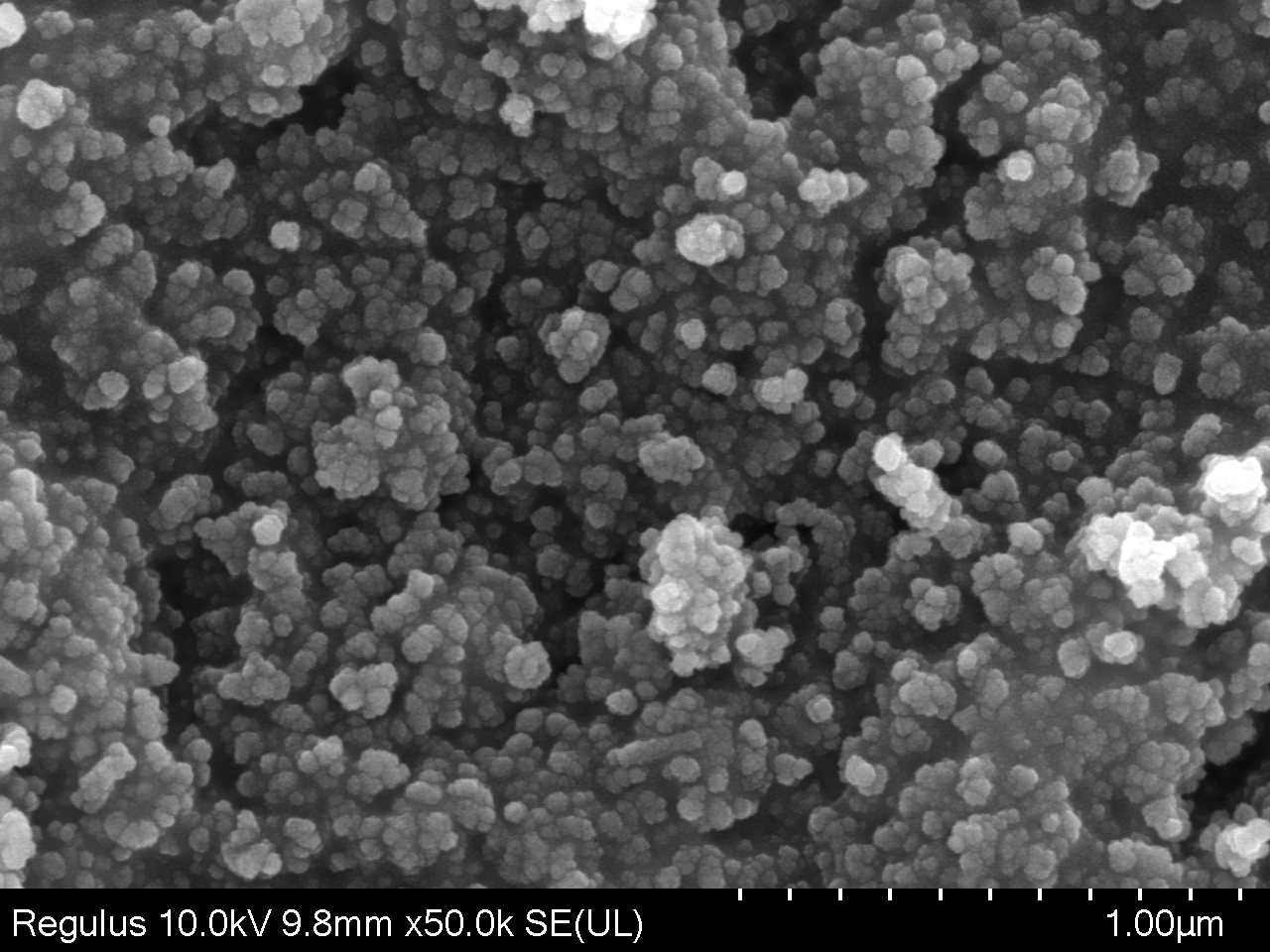

Supplement: S1 Fig — (TIF) [file pone.0321741.s001.tif]

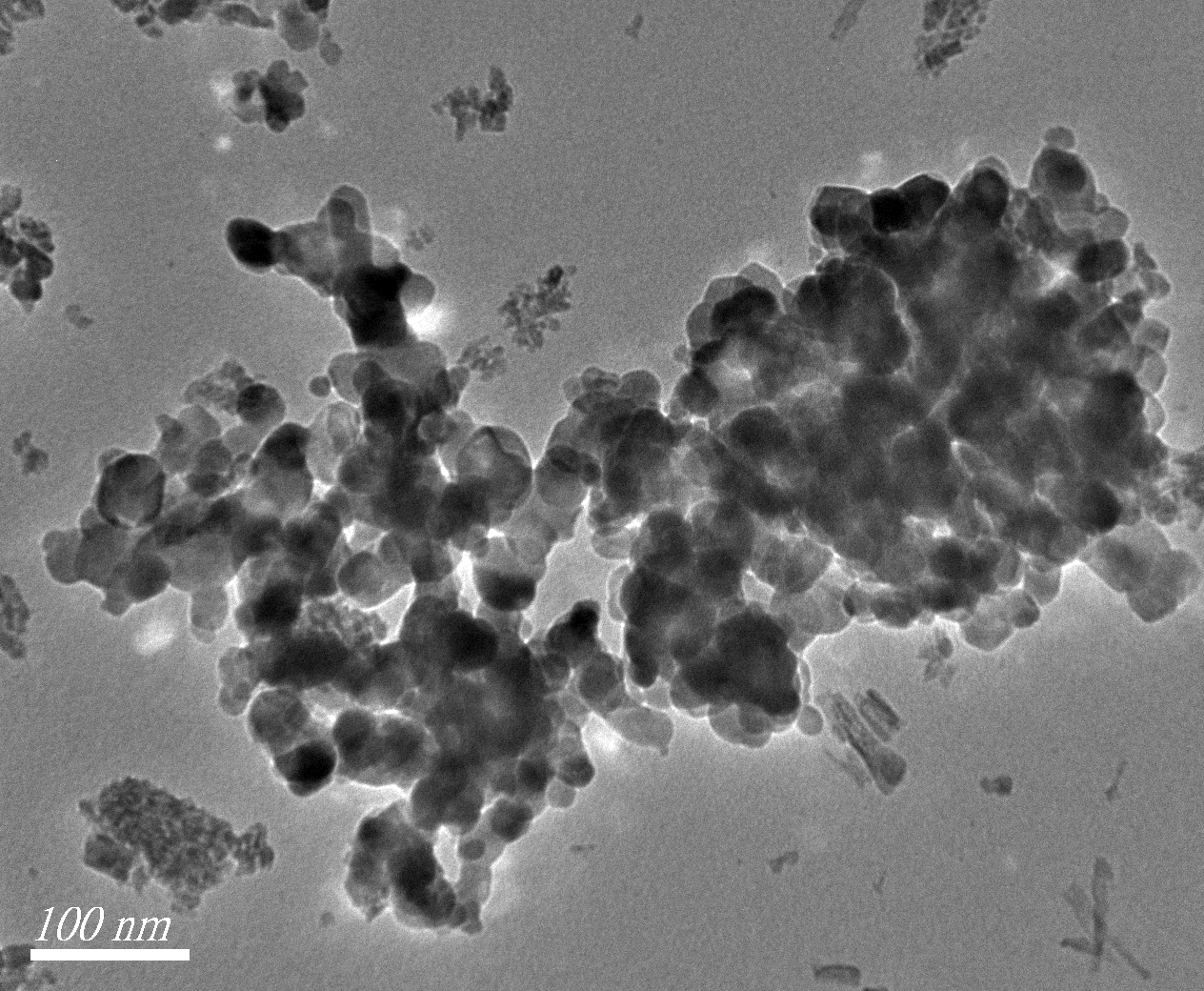

Supplement: S2 Fig — (BMP) [file pone.0321741.s002.bmp]
